# Pulmonary arterial response to *Angiostrongylus vasorum* in naturally infected dogs: echocardiographic findings in two cases

**DOI:** 10.1186/s13071-019-3544-2

**Published:** 2019-06-04

**Authors:** Andrea Corda, Silvia Carta, Antonio Varcasia, Claudia Tamponi, Maria Antonietta Evangelisti, Antonio Scala, Maria Luisa Pinna Parpaglia

**Affiliations:** Dipartimento di Medicina Veterinaria, Università degli Studi di Sassari, Sassari, Italy

**Keywords:** Pulmonary hypertension, Arteriovenous anastomoses, Cardiopulmonary parasites, Angiostrongylosis, Echocardiography

## Abstract

**Background:**

*Angiostrongylus vasorum* is a nematode living in the pulmonary arteries of canids. Infected dogs develop severe pulmonary lesions which can potentially lead to pulmonary hypertension (PH). However, reports of PH in natural infected dogs are scant. One of the possible causes of the low prevalence of PH in *A. vasorum*-infected dogs could be the establishment of large diameter intrapulmonary arteriovenous anastomoses (IPAVAs), which attenuate pulmonary vascular resistance, thus reducing the pulmonary arterial pressure. The present report describes the pulmonary arterial pressure (PAP) response to *A. vasorum* natural infection in two dogs, assessed by echocardiography and by the saline contrast echocardiographic test (SCE).

**Results:**

Both dogs showed clinical signs of respiratory disease. At presentation, case 1 did not show echocardiographic signs of PH and the SCE test was positive proving the presence of IPAVAs. However, at the follow-up visit, despite *A. vasorum* infection resolution, the same dog showed PH and the SCE test resulted negative, which ruled out the presence of IPAVAs. Case 2 suffered from severe pulmonary arterial hypertension and right-side congestive heart failure since the day of presentation. Saline contrast echocardiography was negative both at the time of presentation and at the follow-up visit.

**Conclusions:**

In the two cases described above, the PH was not associated with IPAVAs. During *A. vasorum* infection, IPAVAs recruitment mechanism is able to contrast the rise of PAP until a certain level. It probably represents an initial escape mechanism of PH that, over time, exhausts its compensatory capacities allowing PAP to rise and to be detectable on echocardiography.

**Electronic supplementary material:**

The online version of this article (10.1186/s13071-019-3544-2) contains supplementary material, which is available to authorized users.

## Background

*Angiostrongylus vasorum* (Nematoda; Metastrongyloidea) is a nematode living in the right chamber of the heart and pulmonary arteries of wild canids (mainly foxes), dogs and, occasionally, other animals [1]. *Angiostrongylus vasorum* develops in dogs after the ingestion of infective third-stage larvae (L3) that reside in gastropod molluscs such as snails and slugs, which are needed to complete the parasite’s life-cycle. *Angiostrongylus vasorum* is defined as “the great pretender” [2] because it is responsible for different clinical pictures. Moreover, hematological [3] and imaging findings [4, 5] are not specific to *A. vasorum* infection. The clinical signs can vary from an apparently healthy condition to severe clinical manifestations and the most commonly reported clinical signs are related to respiratory, coagulation [6, 7] and neurological disorders [8, 9]. If not treated, *A. vasorum* infection may be progressive and potentially fatal [2, 10]. Dogs infected with *A. vasorum* develop severe pulmonary lesions which can potentially lead to pulmonary hypertension (PH) [11] which is defined as persistent abnormal increase of pulmonary vascular system blood pressure [12]. Despite the widespread distribution of *A. vasorum* infection and the severity of pulmonary lesions, reports of PH in natural infected dogs have been uncommonly described [13, 14]. Some authors have hypothesized that one of the possible causes of the low prevalence of PH in *A. vasorum* infected dogs could be the recruitment of large diameter intrapulmonary arteriovenous anastomoses (IPAVAs) which attenuate pulmonary vascular resistance, reducing the pulmonary arterial pressure (PAP) [15, 16]. The present report describes the pulmonary arterial response to *A. vasorum* natural infection in two dogs, assessed by echocardiography.

## Results

Case 1: First-stage larvae were detected using the Baermann technique and subsequently identified as *A. vasorum* using morphometrical keys as previously described [17]. Biomolecular characterization allowed us to confirm the morphological identification of the first-stage larvae of *A. vasorum* (GenBank: KF270685.1, KF270683.1). The alignment of our sequence with BLAST showed a homology of 100% with the *cox*1 and ITS-2 sequences of *A. vasorum* available in GenBank (EU493165.1 and EU627597.1, respectively). The Knottʼs test and in-clinic *Dirofilaria immitis*, *Anaplasma* spp., *Ehrlichia* spp. and *Borrelia burgdorferi* (*sensu lato*) tests scored negative.

**Fig. 1:**
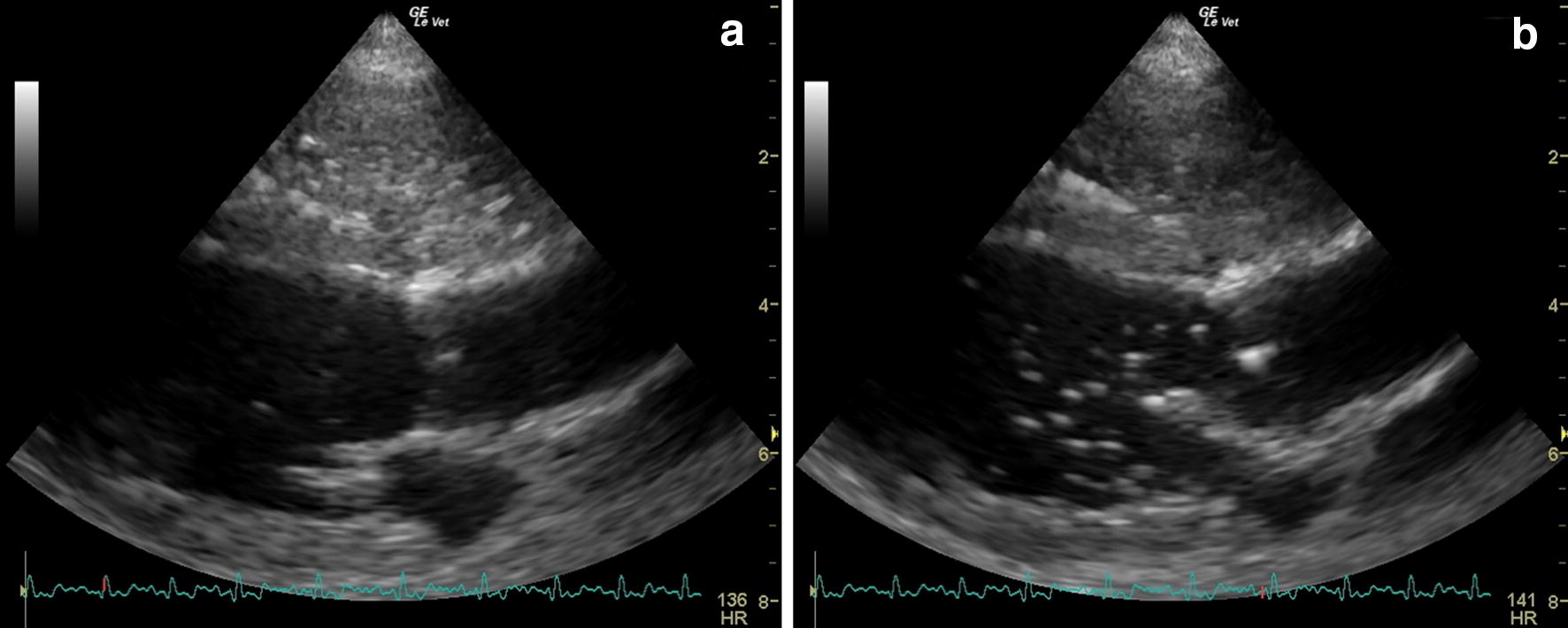
Echocardiographic images during saline contrast test performed in case 1 at the time of presentation (T0). **a** The passage of saline contrast microbubbles in the right ventricle. **b** The passage of the saline contrast microbubbles in the left ventricle 5 cardiac cycles after the appearance in the right heart demonstrating the presence of intrapulmonary arteriovenous anastomosis

Haematology and serum biochemistry profiles did not show abnormalities except for a mild hypergammaglobulinemia and mild neutrophilic leucocytosis. Urinalysis results were normal. Chest radiographies showed a multifocal mixed unstructured interstitial pulmonary pattern. At presentation (T0) echocardiography did not detect signs of PH (Table 1). The saline contrast echocardiography (SCE) test was positive: microbubbles appeared in the left heart 5 cardiac cycles after their appearance in the right heart, proving the presence of IPAVAs (Fig. 1). An additional movie file shows this in more detail (Additional file 1).

**Table 1:** Echocardiographic indirect measurements of pulmonary arterial pressure and ventricular 2D measurements in case 1 at presentation (T0) and at follow-up examination (T1) and in case 2 at the presentation. Numbers in bold represent values outside the reference range

	Case 1 (T0)	Case 1 (T1)	Case 2	Reference range
TR (m/s)	Absent	**3.3**	**4.7**	< 2.8 [49]
TR PG (mmHg)	–	**43.56**	**88.48**	< 30 [49]
PR (m/s)	Absent	Absent	**3.39**	< 2.2 [49]
PR PG (mmHg)	–	–	**46.11**	< 20 [49]
RVIDd (cm)	1.4	1.9	2.4	
LVIDd (cm)	3.6	3.6	3	
LVIDd/RVIDd	2.6	**1.9**	**1.2**	≥ 2 [49]
RVWd (cm)	0.5	0.5	0.7	
LVPWd (cm)	0.8	0.9	0.8	
LVPWd/RVWd	1.6	1.6	1	≥ 2 [50]
PA (cm)	2	2	2.3	
Ao (cm)	2	2.2	2	
PA/Ao	1	**0.9**	**1.15**	< 0.98 [47]
RPAD (%)	46	**20**	**4**	> 34.6 [48]
AT (ms)	86	72	37	> 0.80 [47]
ET (ms)	161	185	135	
AT/ET	0.53	**0.38**	**0.27**	> 0.44 [47]
PV (m/s)	0.98	1	0.9

**Fig. 2:**
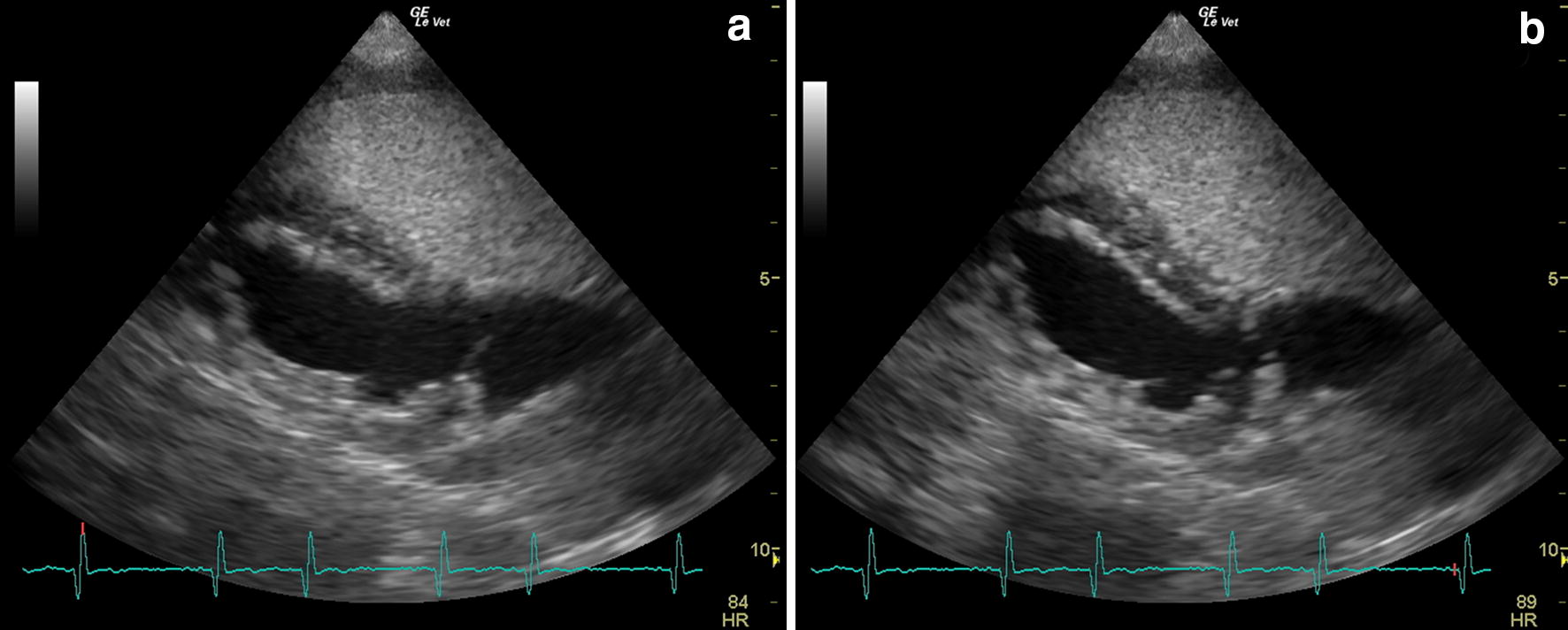
Echocardiographic images during saline contrast test performed in case 2 at time of presentation (T0). **a** The passage of saline contrast microbubbles in the right ventricle. **b** The absence of saline contrast microbubbles in the left heart, ruling out the presence of intrapulmonary arteriovenous anastomosis

Case 2: As in case 1, the parasitological examination identified *A. vasorum* first-stage larvae at morphological and molecular characterization. The Knottʼs test and in-clinic *D. immitis*, *Anaplasma* spp., *Ehrlichia* spp. and *B. burgdorferi* (*s.l.*) test scored negative. A *Leishmania infantum* immunofluorescence antibody test (IFAT) was negative. Blood test results revealed mild normochromic and normocytic anaemia, mild hypergammaglobulinemia and mild increase of alanine aminotransferase and alkaline phosphatase. Urinalysis results were normal. The radiological examination of the thorax revealed generalized cardiomegaly with marked right heart enlargement, moderate bulging of the main pulmonary artery and slight distension of pulmonary arteries. Lateral views showed dorsal bowing of trachea cranial to carina and caudal heart border more vertical than normal. In addition, there was diffuse unstructured interstitial pattern in the lung parenchyma, most apparent in the caudo-dorsal region, and moderate pleural effusion. Abdominal ultrasonography detected moderate ascites, hepatomegaly and hepatic vein dilation. The peritoneal effusion was sampled and analysed; it was compatible with high-protein transudate. Doppler echocardiography revealed signs of severe PH such as an RV-to-RA gradient of 88.48 mmHg, PA-to-RV gradient of 46.11 mmHg and reduction of the AT/ET ratio (Table 1). Moreover, two-dimensional (2D) echocardiography showed RV, RA and PA dilation and reduction of the RPAD index (Table 1). M-mode examination of the LV evidenced paradoxical septal motion and reduction of the LVID in systole and in diastole, suggestive of preload reduction (Table 2). The PA systolic flow profile appeared asymmetric with a rapid acceleration phase and slower deceleration phase. Pulmonic stenosis was ruled out because of normal pulmonic valve leaflets, laminar flow and normal systolic flow velocity. The SCE test was negative: microbubbles did not appear in the left heart following their appearance in the right heart, ruling out the presence of IPAVAs (Fig. 2). An additional movie file shows this in more detail (Additional file 2).

**Table 2:** M-mode measurements and relative normalized values the left ventricular diameter wall thickness in case 1 at presentation (T0) and at follow-up examination (T1) and case 2 at the presentation. Numbers in bold represent values outside the reference range

	Case 1 (T0) 18 kg	Case 1 (T1) 20 kg	Case 2 19 kg	Reference range 95th percentile [51]
IVSd (cm)	1	1	0.9	
IVSd norm	0.5	0.49	0.44	0.33–0.52
LVIDd (cm)	3.5	3.6	3	
LVIDd norm	1.5	1.49	**1.26**	1.35–1.73
LVPWd (cm)	1	0.9	0.8	
LVPWd norm	0.51	0.45	0.4	0.33–0.53
IVSs (cm)	1.4	1.2	0.8	
IVSs norm	0.7	0.58	**0.39**	0.48–0.71
LVIDs (cm)	1.6	2.2	1.7	
LVIDs norm	**0.64**	0.86	**0.67**	0.79–1.14
LVPWs (cm)	1.5	1.6	1	
LVPWs norm	0.77	**0.8**	**0.51**	0.53–0.78

### Treatment

Both dogs underwent medical treatment with fenbendazole (Panacur®, MSD Animal Health) at 50 mg/kg every 24 h for 15 days. In addition, case 1 received amoxicillin-clavulanic acid (Synulox®, Zoetis S.r.l.) 12.5 mg/kg every 12 h for 8 days. In case 2, the right-side congestive heart failure and PH symptoms were treated with sildenafil citrate (Viagra®, Pfizer) at 2 mg/kg every 12 h, benazepril (Fortekor®, Novartis) 0.25 mg/kg every 12 h and furosemide (Diuren®, Teknofarma S.p.A) at 1 mg/kg every 12 h.

### Outcome and follow-up

**Fig. 3:**
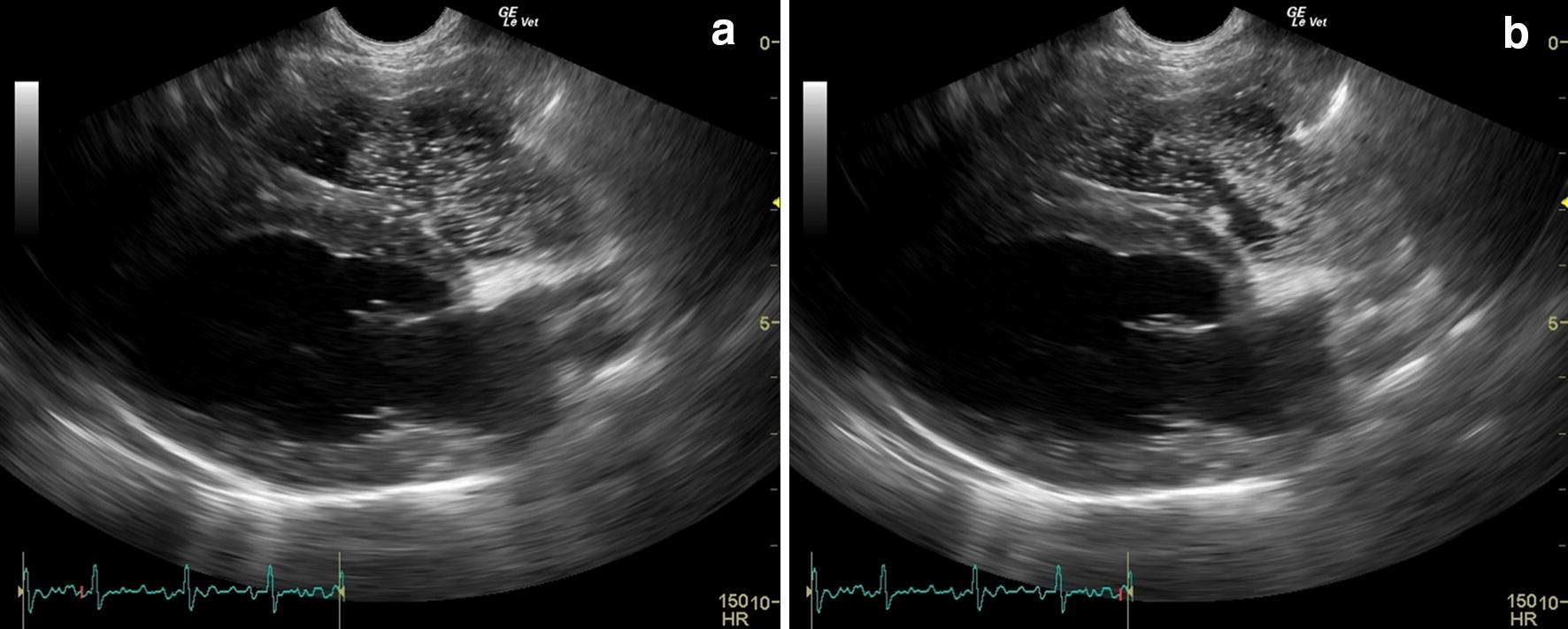
Echocardiographic images during saline contrast test performed in case 1 eight months after presentation (T1). **a** The passage of saline contrast microbubbles in the right ventricle. **b** The absence of saline contrast microbubbles in the left heart, ruling out the presence of intrapulmonary arteriovenous anastomosis at T1

Case 1: Four weeks after treatment with fenbendazole, a coprological follow-up was performed to verify the effectiveness of the treatment; no *A. vasorum* larvae were detected in the Baermann examination. The dog underwent monthly clinical check-ups. Each clinical examination was normal except for the constant presence of abnormal lung sounds (inspiratory crackles), more appreciable in the left lung. Eight months after the presentation (T1) a mild systolic heart murmur, grade II/VI, was detected in the right hemithorax. Then, the dog underwent echocardiography, thoracic radiology, blood and urine analysis and parasitological examination. Parasitological examination at T1 was negative to *A. vasorum* infection, the radiographic bronco-interstitial pattern was less evident than at T0, blood and urine parameters were within the normal limits, echocardiography revealed signs of mild PH characterized by the presence of TR jet of 3.3 m/s (RV-RA systolic gradient: 44 mmHg), mild RV dilation and reduced AT/ET ratio (Table 1). At T1, SCE was negative because no microbubbles were observed in the left heart after they had arrived in the RV, ruling out the presence of IPAVAs (Fig. 3). An additional movie file shows this in more detail (Additional file 3).

Case 2: Initially the dog displayed clinical improvement. Four weeks after fenbendazole treatment, the dog underwent a follow-up visit and all the exams were repeated. The copromicroscopical examination by the Baermann method did not evidence *A. vasorum* larvae. The echocardiographic examination did not differ significantly from the previous one, still showing severe PH. The SCE test was still negative: microbubbles did not appear in the left heart following their appearance in the right heart, ruling out the presence of IPAVAs. Unfortunately, 3 months after presentation, the dog was euthanized because of refractory right-sided congestive heart failure. The owner denied consent for the anatomopathological examination.

## Discussion

To the best of our knowledge, these are the first descriptions of clinical cases of *A. vasorum* in dogs from Sardinia island (Italy), where the parasite was reported for the first time by Pipia et al. [18]. Recent surveys demonstrate an increasing incidence of angiostrongylosis in different European countries [19, 20] and its spread in previously non-endemic areas such as the Netherlands, Germany and Greece [21–23]. The first case report of canine angiostrongylosis in Italy was described in 2002 by Della Santa et al. [24]. Recently, new cases have been reported in different Italian regions [25] with the highest prevalence in the north and in the central part of the country [26] where there are ideal climatic conditions for the maintenance and development of the intermediate hosts [19].

Dogs infected with *A. vasorum* develop severe pulmonary lesions, secondary to the intense immune response to eggs and larval antigens, including granulomatous inflammation, haemorrhage, arterial thrombosis, periarteritis and interstitial fibrosis [14, 27, 28]. Pulmonary injuries induced by *A. vasorum* infection are determinants for the development of PH [29]. Chronic PH can lead to right-sided congestive heart failure, as seen in case 2. Despite the severity of pulmonary lesions, PH is reported only in 14.6% of dogs infected with *A. vasorum* [11]. A study conducted by Kranjc et al. [14] evidenced that 13 weeks post-experimental *A. vasorum* infection, 80% of the lungs were consolidated. Thrombi associated with parasitic larvae were present in the pulmonary arteries of untreated dogs but, at the same time point, echocardiographic-derived PAP estimation was within the normal limits. Matos et al. [15, 16] hypothesized that one of the possible causes of the low prevalence of PH in *A. vasorum* infected dogs could be the recruitment of large diameter IPAVAs. The IPAVAs are dynamic preformed vascular conduits that have the function of avoiding excessive flow and pressure increases in the pulmonary microcirculation during exercise [30], following acute pulmonary embolism [31] and in response to hypoxia [32] in order to minimize the negative effects of PH on the capillary bed and reduce RV afterload [16]. Large diameter IPAVAs are known to exist in many species including humans [33, 34] and dogs [15, 16, 31]. The presence of IPAVAs can be demonstrated by the SCE test [15, 16, 34, 35]. Normally, agitated saline solution injected peripherally is entirely removed by the lungs microcirculation [36]. The appearance of contrast in the left heart, not earlier than three cardiac cycles following contrast opacification of the RV is indicative of the presence of IPAVAs [15, 16, 37]. In the two cases described above, the PH was not associated with IPAVAs. Case 2, at presentation and at follow-up visit, showed symptoms and echocardiographic findings of severe PH and the SCE test was negative, ruling out the presence of IPAVAs. In this dog, the PH never reverted after treatment, even though *A. vasorum* was eliminated. This may have been due to the irreversibility of the pulmonary vascular or parenchymal lesions induced by *A. vasorum.* Conversely, case 1, at T0, did not show symptoms or echocardiographic signs of PH and the SCE test revealed the presence of IPAVAs. The same dog eight months later (T1), despite being successfully treated against *A. vasorum* infection, showed echocardiographic signs of mild PH and the SCE test was negative. These results may have been due to the fibrotic evolution of the *A. vasorum* lesions and/or to progression of lesions caused by *A. vasorum* death after treatment. Whatever the cause, we hypothesized that the increase of PAP may have induced the closure of the IPAVAs with the appearance of PH. An alternative hypothesis is that IPAVAs may be localized in areas of the pulmonary circulation that are recruited under conditions of elevated pulmonary blood flow and pressure [38]. If true, then a reduction in blood flow through IPAVAs may indicate the loss of these areas of the lungs because of the pulmonary lesions secondary to *A. vasorum* infection. Our findings are consistent with those of Matos et al. in dogs naturally [16] and experimentally [15] infected with *A. vasorum.* They observed that dogs with negative SCE results had higher PAP [15, 16]. The authors of those studies hypothesized that some dogs were able to open the IPAVAs and therefore to counteract the PAP increase while others did not. Based on our SCE and echocardiographic results we speculated that the IPAVAs recruitment mechanisms are able to contrast the rise of PAP until a certain level, and it probably represents an initial escape mechanism of PH that over time exhausts its compensatory capacities allowing PAP to rise and to be detectable on echocardiography. In both clinical cases described in this report, we hypothesize that PH was secondary to *A. vasorum* infection. Other causes of PH, such as congenital or acquired heart diseases [12], were excluded by the echocardiographic examination. Heartworm disease was ruled out by the Knottʼs test and by the in-clinic heartworm antigen test. Interestingly, the cross reaction between *A. vasorum* and *D. immitis* was not detected, confirming the results obtained by Schnyder et al. [39] who did not detect cross-reactions between the two parasites using the same antigen test used in the present survey. Based on the clinical findings and laboratory results, we consider the presence of PH secondary to pulmonary thromboembolism caused by other systemic diseases [12] as unlikely. Several treatment options have been proposed against *A. vasorum* infection [40, 41], but in our patients we decided to use fenbendazole at 50 mg/kg per day, for 15 days. In both cases the treatment was effective in eliminating *A. vasorum* from the infected dogs.

## Conclusions

This report confirms that echocardiography associated with the SCE test are two valid methods for assessing and monitoring the PAP response to *A. vasorum* infection in naturally infected dogs. However, the precise role played by IPAVAs in the pathophysiology of PH during angiostrongylosis requires further investigation.

## Methods

### Clinical examination

Case 1 was 5 years-old, female, neutered, 18 kg hunting dog, guested in a public kennel in Sassari (Italy). The dog resulted positive to *A. vasorum* infection during a parasitological faecal screening examination. Three months before, the dog started a treatment against leishmaniasis infection with oral miltefosine (Milteforan®, Virbac S.r.l.) at 2 mg/kg/day for 28 days and oral allopurinol (Zyloric®, Teofarma S.r.l.) at 10 mg/kg every 12 h for six months. Then, at the time of presentation the dog was still on therapy with allopurinol. At presentation (T0), the dog was bright, alert and responsive. The physical examination evidenced an increase in respiratory rate (40 beats per minute) and the presence of moderate lung inspiratory crackles, localized in the left caudal lung. The dog did not present clinical signs relative to neurological or coagulation disorders. The kennel’s employees did not report symptoms related to respiratory disease (e.g. cough).

Case 2 was a 10 years-old, female, neutered, 19 kg dog, presented because of exercise intolerance, chronic cough, dyspnoea, weight loss and abdominal distension. The dog did not present symptoms related to neurological or coagulation disorders. On physical examination the dog showed tachypnoea, abdominal distension, pale mucous membranes, jugular vein pulse and distension, right systolic heart murmur (grade III/VI) and severe crackle sounds diffused on both lungs.

Each dog underwent parasitological examination, blood analysis, chest radiography, standard echocardiography and a saline contrast echocardiography (SCE) test.

### Parasitological examination

The Baermann technique was performed as previously described [18, 42]. Subsequently, larvae were examined and measured under the light microscope. Larvae were identified using morphometrical keys for metastrongyles of dogs [17] and DNA was extracted from positive sediment containing L1 larvae with a commercial kit (High Pure PCR Template Preparation kit, Roche Diagnostics, Mannheim, Germany). Molecular characterization was performed for the mitochondrial cytochrome *c* oxidase subunit 1 (*cox*1) and for the second ribosomal transcribed spacer region (ITS-2) of the ribosomal RNA gene as previously described [43, 44]. PCR products were then purified using a ChargeSwitch® PCR Clean-Up Kit (Invitrogen, Carlsbad, California, USA) and sequenced (MWG Biotech/M-Medical, Milan, Italy). Sequences were then aligned and compared with those available in GenBank™ using BLAST analysis. The blood was tested also for *D. immitis*, *Anaplasma* spp., *Ehrlichia* spp. and *B. burgdorferi* (*s.l.*) using an ELISA in-clinic antigen test (Snap 4DX, IDEXX Laboratories, Westbrook, ME, USA). Blood samples were also analyzed using Knott’s technique as previously described [18]. Finally, sera samples were tested with an IFAT for *L. infantum.*

### Blood analysis

Blood analysis was performed using a clinical chemistry analyser (ABX Pentra 400, Horiba Medical, Kyoto, Japan) and an automated haematology analyser (Lasercyte, IDEEX Laboratories). Serum protein electrophoresis and urinalysis were performed at an external laboratory (Ekosistems Srl, Sassari, Italy). Coagulation tests were not performed.

### Chest radiography

Thoracic radiographs were obtained within 24 h of the echocardiographic examination; both dogs underwent right lateral and ventro-dorsal views. Thoracic radiographs were evaluated by an experienced veterinary radiologist.

### Echocardiography

Echocardiographic exams were performed by a single experienced operator with a portable ultrasound unit (LOGIQ e, General Electric, Boston, Massachussets, USA) equipped with a multifrequency (1.5–3.5 MHz) phased array transducer (3S-RS) and a multi frequency (4–11 MHz) microconvex (8C-RS) probe. The unsedated dogs were positioned alternately in right and left lateral recumbence on a raised table with a central opening, designed for veterinary echocardiographic examination. A complete transthoracic echocardiographic examination, including 2D, M-mode and Doppler, was performed on each dog, according with previously published guidelines [45]. The primary focus of the echocardiographic examination was to indirectly estimate the PAP. In the presence of tricuspid (TR) and/or pulmonary regurgitation (PR), the Doppler-derived peak regurgitant flow velocity was measured and used to estimate systolic and diastolic PAP, respectively, from the simplified Bernoulli equation [46]. Moreover, from the B-mode echocardiography we considered the main pulmonary artery/aorta ratio (PA/Ao) [47], the right pulmonary artery distensibility index (RPAD) [48], the right ventricular (RV) chamber size and the RV wall thickness. The RV diastolic diameter (RVIDd) was considered normal if it was less than or equal to one half of the size of the left ventricle diastolic diameter (LVIDd) in the right parasternal long-axis view of the LV outflow tract. The RV was considered moderately dilated if it was from 50 to 100% of LV, and severe dilatation was reported when the RV size exceeded that of the LV [49]. The RV wall thickness in diastole (RVWd) was considered hypertrophic if greater than half of the LV posterior wall in diastole (LVPWd), in the right parasternal long-axis LV outflow view [50]. With the pulsed wave Doppler, acquired from the right parasternal short-axis view at the level of the heart base, we evaluated the PA systolic flow profile and measured the RV systolic time intervals, including the pulmonary flow acceleration time (AT), ejection time (ET) and AT/ET ratio [47–49]. Left ventricular wall thickness and internal diameter were also measured by M-mode echocardiography and results were normalized to body weight (BW) as previously described [51]. Other echocardiographic evidence of PH such as right atrial (RA) dilation, presence of paradoxical septal motion, PA flow profile abnormalities and reduction in LV chamber size [12] were evaluated. All images and loops were stored and analysed offline by the same operator. Each echocardiographic measurement was repeated on three consecutive cardiac cycles, and mean values were calculated. The measured echocardiographic parameters and the reference values are listed in Table 1. Saline contrast echocardiography was performed from the right parasternal long-axis 4-chamber view. The presence of intrapulmonary IPAVAs was assessed by the clinical methodology described by Matos et al. [16]: the SCE test was defined positive if microbubbles appeared in the left heart not earlier than 3 cardiac cycles following contrast opacification of the right heart.

## Additional files

**Additional file 1.** Echocardiographic cine-loop of saline contrast test performed in case 1 at the time of presentation (T0). The video shows the appearance of saline contrast microbubbles in the left heart following their passage in the right heart, demonstrating the presence of intrapulmonary arteriovenous anastomosis.

**Additional file 2.** Echocardiographic cine-loop of saline contrast test performed in case 2 at the time of presentation. The video shows the passage of saline contrast microbubbles in the right ventricle. Microbubbles did not appear in the left heart following their appearance in the right heart, ruling out the presence of IPAVAs.

**Additional file 3.** Echocardiographic cine-loop of saline contrast test performed in case 1 eight months after presentation (T1). The video shows the passage of saline contrast microbubbles in the right ventricle. Microbubbles did not appear in the left heart following their appearance in the right heart, ruling out the presence of IPAVAs.

## Data Availability

All relevant data supporting the conclusions of this article are included within the article and its additional files. Newly generated sequences were deposited in the GenBank database with the Accession Codes KF270685.1 and KF270683.1.

## Abbreviations

2D: two-dimensional echocardiography
Ao: aorta
AT: acceleration time
BLAST: Basic Local Alignment Search Tool
ECG: electrocardiogram
ET: ejection time
IFAT: immunofluorescence antibody test
IPAVAs: intrapulmonary arteriovenous anastomoses
LV: left ventricle
LVIDd: left ventricular internal diameter in diastole
LVWd: left ventricular wall in diastole
PA: pulmonary artery
PAP: pulmonary arterial pressure
PH: pulmonary hypertension
PR: pulmonary regurgitation
RA: right atrium
RPAD: right pulmonary artery distensibility index
RV: right ventricle
RVIDd: right ventricle internal diameter in diastole
RVWd: right ventricular wall in diastole
SCE: saline contrast echocardiography
TR: tricuspid regurgitation
